# Collateral Damage: Ruptured Peripancreatic Pseudoaneurysm Associated With Severe Celiac Artery Stenosis From Median Arcuate Ligament Compression

**DOI:** 10.7759/cureus.113649

**Published:** 2026-07-30

**Authors:** Wesam Soliman, Emily N Rados, Rawad Daniel Arja, Erwin Tabucanon, Daniel Ortolano

**Affiliations:** 1 Surgery, Valley Health System, Las Vegas, USA; 2 Internal Medicine, Valley Health System, Las Vegas, USA; 3 Internal Medicine, United Health Services, Las Vegas, USA

**Keywords:** celiac stenosis, coil embolization, hemoperitoneum, median arcuate ligament syndrome, mesenteric collateral, pancreaticoduodenal artery, peripancreatic bleeding, pseudoaneurysm, thrombin injection, visceral aneurysm

## Abstract

Median arcuate ligament syndrome (MALS) results from compression of the celiac artery by the median arcuate ligament and is often asymptomatic or associated with chronic postprandial abdominal pain. Rarely, severe celiac artery stenosis can lead to increased collateral flow through the pancreaticoduodenal arcade and life-threatening hemorrhagic complications. We report the case of a 57-year-old woman with no history of pancreatitis, trauma, or abdominal surgery who presented with acute abdominal pain and was found to have a ruptured peripancreatic pseudoaneurysm arising from a celiac-superior mesenteric artery (SMA) collateral vessel. Computed tomography angiography (CTA) demonstrated severe proximal celiac artery stenosis with a hook-shaped narrowing, with the collateral vessel arising from the pancreaticoduodenal arcade, suggesting MALS. After an unsuccessful initial angiographic attempt, repeat intervention required direct percutaneous access, the use of thrombin injection to control active extravasation, and coil embolization of the pseudoaneurysm's inflow and outflow vessels. Her course was complicated by hemorrhagic shock, respiratory failure, and pulseless electrical activity arrest with return of spontaneous circulation. This case highlights an unusual life-threatening presentation of suspected MALS-related collateral vessel injury.

## Introduction

Median arcuate ligament syndrome (MALS), also known as Dunbar syndrome, is caused by extrinsic compression of the celiac artery by the median arcuate ligament. Although celiac artery compression may be seen incidentally on imaging, symptomatic MALS is uncommon; MALS classically presents with postprandial epigastric pain, nausea, vomiting, weight loss, or an abdominal bruit [1].

In severe celiac artery stenosis, collateral flow from the superior mesenteric artery (SMA) may increase through the pancreaticoduodenal arcade to supply the celiac territory [2,3]. This altered hemodynamic pathway can become clinically significant when enlarged collateral vessels develop vascular complications, including pseudoaneurysm formation and rupture. These complications are rare but may present with acute abdominal pain, retroperitoneal or intraperitoneal hemorrhage, and hemodynamic instability.

We present a rare case of a ruptured peripancreatic pseudoaneurysm arising from a celiac-SMA collateral vessel in a patient with suspected MALS in the absence of common predisposing factors such as pancreatitis, trauma, or prior abdominal surgery. The patient required a complex endovascular rescue approach after an unsuccessful initial angiographic attempt, highlighting an uncommon and life-threatening vascular complication of MALS.

## Case presentation

A 57-year-old woman with a history of hypothyroidism presented to the emergency department with one day of acute right-sided abdominal pain associated with nausea, vomiting, and diarrhea. She denied any prior history of pancreatitis, abdominal trauma, or abdominal surgery. On arrival, she was found to be in atrial fibrillation with rapid ventricular response and was started on amiodarone. Initial laboratory evaluation showed leukocytosis of 15.47 × 10³/μL, hyperglycemia of 14.7 mmol/L, sodium of 134 mmol/L, potassium of 3.2 mmol/L, anion gap of 18 mmol/L, calcium of 2.12 mmol/L, total bilirubin of 19.0 μmol/L, and lactic acid of 8.4 mmol/L. Her initial hemoglobin was 11.8 g/dL (Table 1).

**Table 1:** Patient's laboratory values on admission

Parameter	Patient value	Reference range
Hemoglobin	11.8 g/dL	10.7-16.7 g/dL
WBC count	15.47 × 10³/μL	3.74-10.90 × 10³/μL
Glucose level	14.7 mmol/L	74-106 mmol/L
Sodium	134 mmol/L	136 -145 mmol/L
Potassium	3.2 mmol/L	3.5-5.1 mmol/L
Anion gap	18 mmol/L	5-14 mmol/L
Calcium	2.12 mmol/L	2.17-2.6 mmol/L
Total bilirubin	19.0 μmol/L	3.42-17.1 μmol/L
Lactic acid	8.4 mmol/L	0.5-2.2 mmol/L

**Figure 1:**
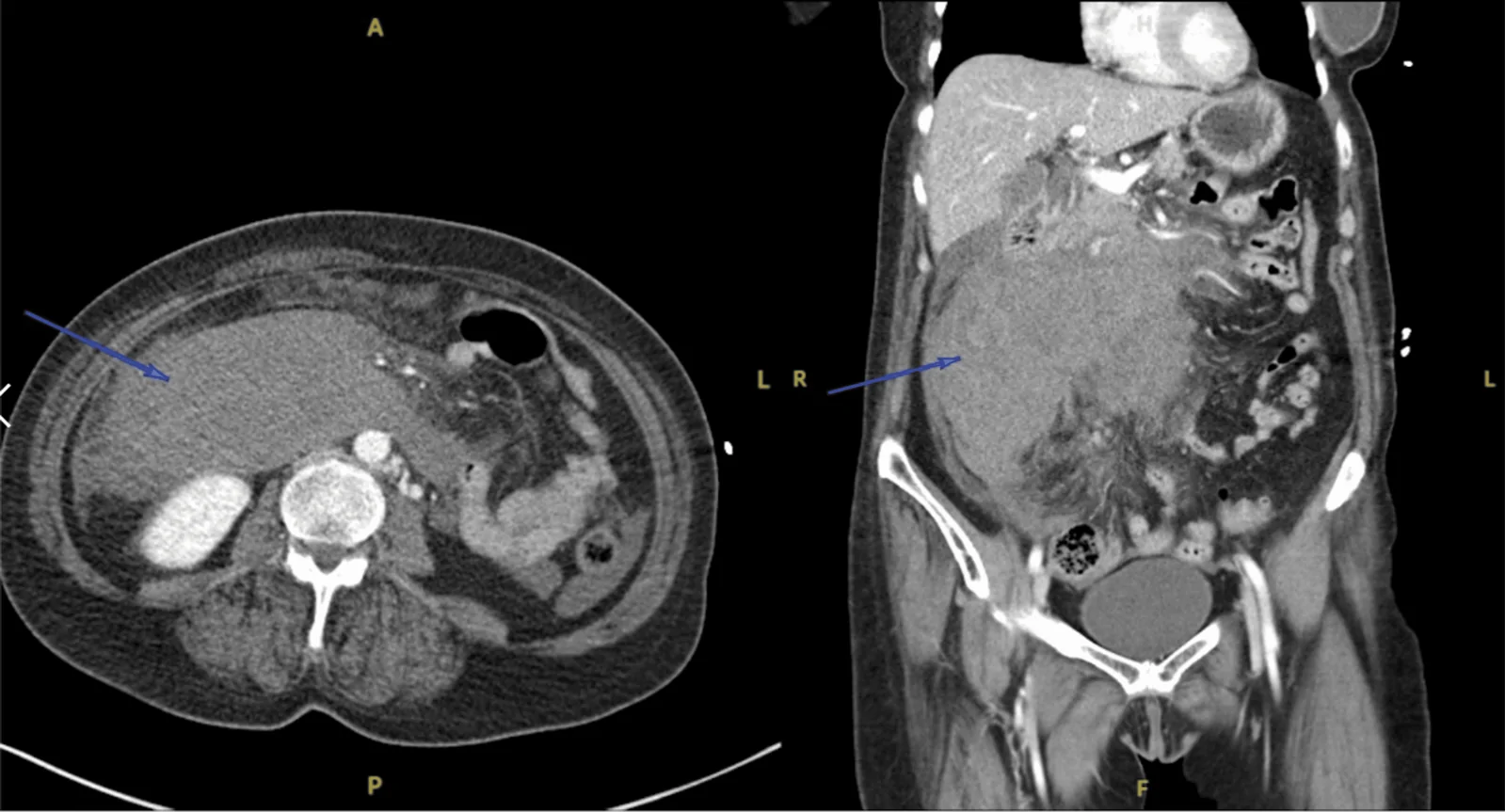
Initial contrast-enhanced CT demonstrated a large right hemiabdominal hematoma/fluid collection with surrounding inflammatory stranding and regional mass effect. The blue arrows point to a large mass-like structure in the right hemiabdomen with marked inflammatory stranding, likely a large fluid collection in the coronal view (left) and axial view (right).

Contrast-enhanced CT of the abdomen and pelvis demonstrated a large mass-like structure in the right mid-to-lower abdomen measuring approximately 12.5 x 8.6 x 14.3 cm with marked inflammatory stranding. There was mild peripancreatic edema and regional bowel wall thickening involving the hepatic flexure and ascending colon. An indeterminate focus of contrast was seen within the region of inflammatory change and fluid areas, raising concern for vessel involvement, thrombus, or pseudoaneurysm. No gross free air was identified. The initial differential diagnosis included hemorrhage, infected fluid collection, complex pancreatic fluid collection, bowel pathology, and vascular abnormality (Figure 1). 

On hospital day two, leukocytosis persisted at 15.01 × 10³/μL, and the patient's hemoglobin decreased from 11.8 g/dL to 8.5 g/dL. Mesenteric angiography was performed, including SMA angiography, celiac arteriography, common hepatic artery angiography, gastroduodenal artery angiography, and gastroepiploic artery angiography. The study demonstrated numerous tortuous celiac-mesenteric collaterals in the peripancreatic region and a 13 x 13 mm peripancreatic pseudoaneurysm arising from a branch of the inferior pancreaticoduodenal artery. There was retrograde flow within the common hepatic and gastroduodenal arteries. The feeding vessel could not be identified or cannulated despite prolonged attempts, and embolization was aborted. No active extravasation was seen during this procedure.

**Figure 2:**
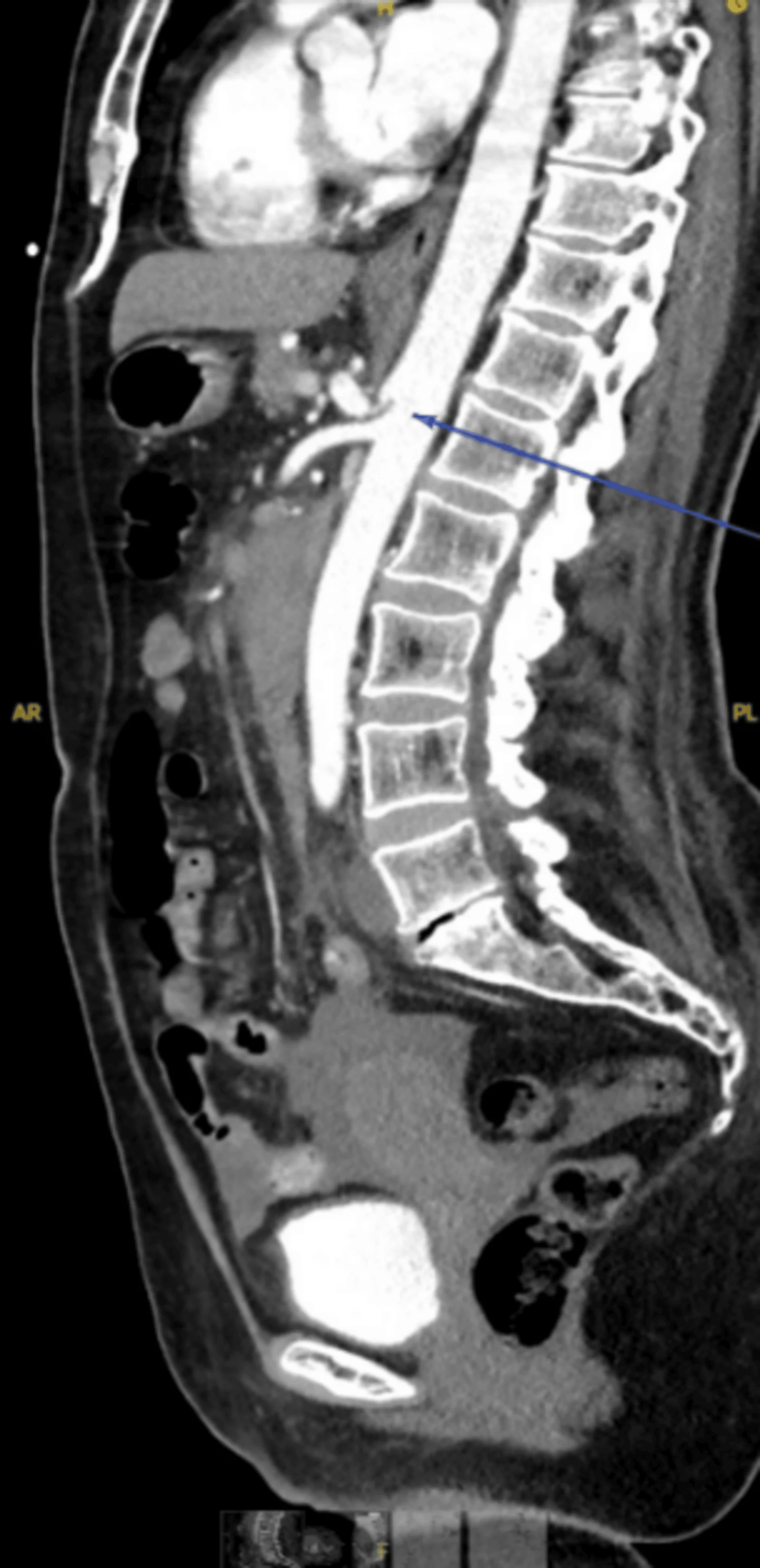
Sagittal view of the CTA revealed severe focal narrowing with a hook-shaped configuration of the proximal celiac artery (blue arrow), supporting the diagnosis of MALS. CTA: Computed tomography angiography, MALS: Median arcuate ligament syndrome

On hospital day three, the patient continued to experience right-sided abdominal pain, and her hemoglobin further decreased to 5.8 g/dL. Two units of packed RBCs were ordered, and a stat computed tomography angiography (CTA) of the abdomen and pelvis was obtained. This demonstrated a 9 x 9 mm peripancreatic pseudoaneurysm arising from a peripancreatic celiac-SMA collateral vessel. There was an associated large abdominal hematoma measuring approximately 14 x 5.6 cm, similar to the prior study, but pelvic hemoperitoneum had increased. Severe stenosis of the proximal celiac artery with significant celiac-SMA collateralization was also identified. Sagittal imaging demonstrated a hook-shaped narrowing of the proximal celiac artery, supporting MALS (Figure 2). 

**Figure 3:**
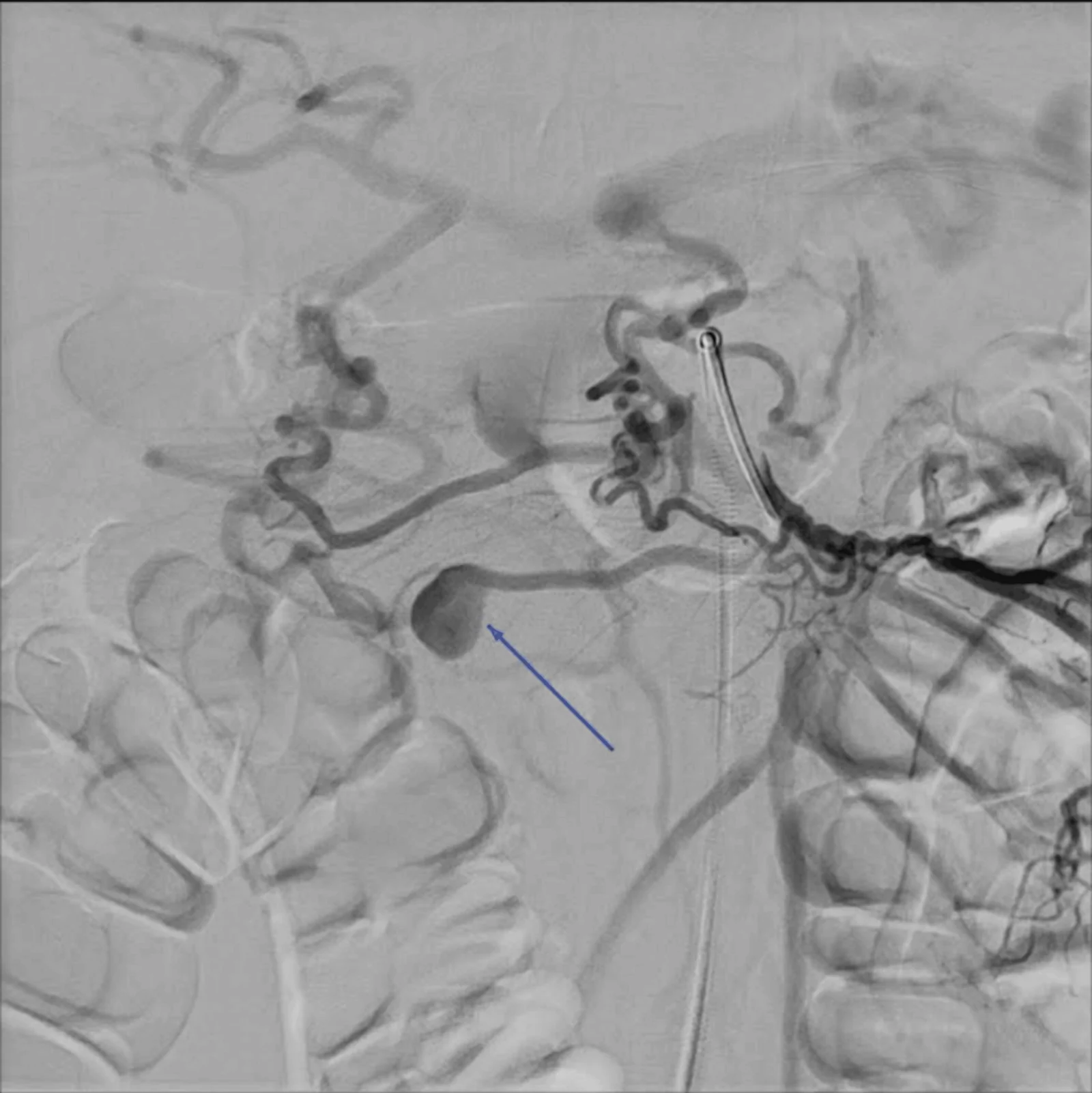
Mesenteric angiography demonstrated prominent tortuous celiac-superior mesenteric artery collateralizations through the pancreaticoduodenal arcade The blue arrow indicates a contrast-filled peripancreatic pseudoaneurysm.

The patient was emergently taken for repeat interventional radiology (IR) treatment. Angiography confirmed very severe stenosis at the origin of the celiac artery, marked peripancreatic celiac-SMA collateralization, and a pseudoaneurysm at the junction between the superior and inferior pancreaticoduodenal arteries (Figure 3). 

**Figure 4:**
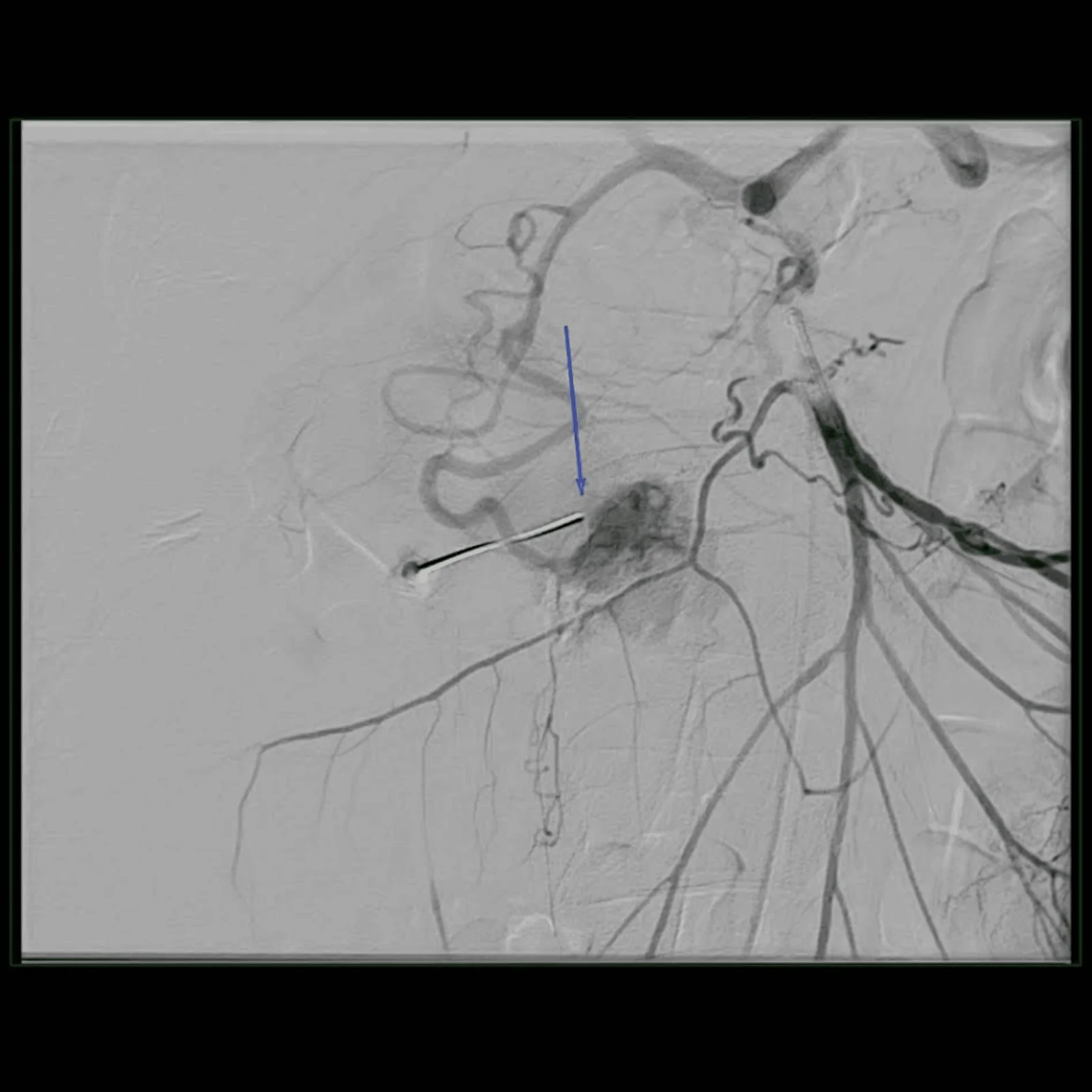
Angiographic image during direct percutaneous access of the pseudoaneurysm The blue arrow indicates the pseudoaneurysm/collateral region treated with direct thrombin injection for active extravasation.

Initial attempts to cannulate the feeding branch transarterially were unsuccessful because of the tortuous collateral vascular anatomy. The pseudoaneurysm was then accessed directly by percutaneous puncture under angiographic guidance. During direct access, active extravasation from the pseudoaneurysm was observed, and 1,000 units of thrombin were injected directly into the pseudoaneurysm sac to control bleeding (Figure 4).

**Figure 5:**
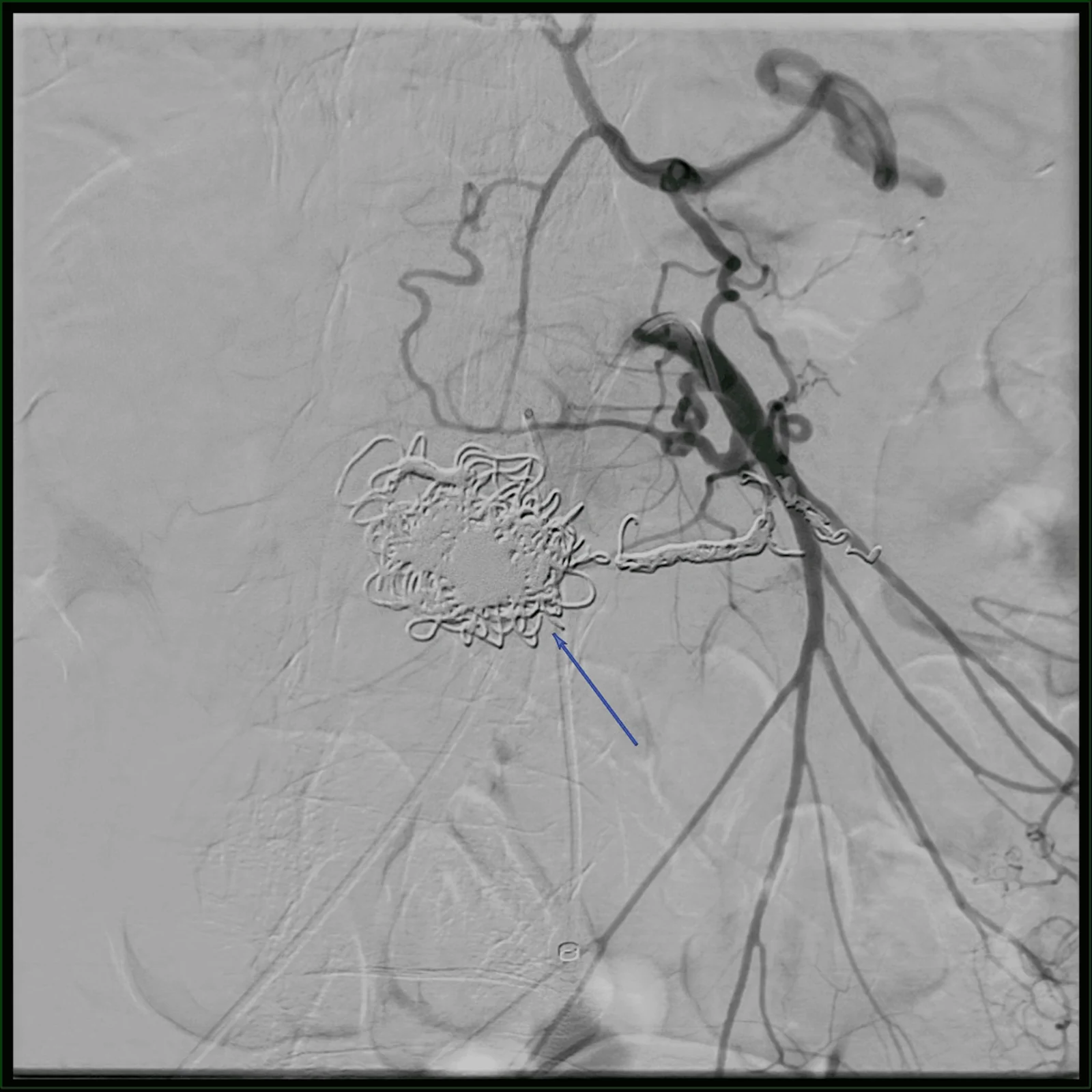
Completed angiography after coil embolization demonstrated the coil packed with preserved major mesenteric arterial flow The blue arrow indicates the coil pack in the treated pseudoaneurysm region.

The proximal jejunal branch supplying the pseudoaneurysm was then embolized with coils. The superior pancreaticoduodenal artery outflow was embolized with detachable coils, followed by coil embolization of the pseudoaneurysm and inferior pancreaticoduodenal artery inflow. After the procedures, angiography demonstrated complete exclusion of the pseudoaneurysm with preserved flow in the SMA, common hepatic artery, and gastroduodenal artery (Figure 5).

Shortly after the embolization procedure, the patient developed hemodynamic collapse with systolic blood pressure in the 60s, consistent with hemorrhagic shock. She required norepinephrine support and activation of the massive transfusion protocol (MTP), receiving six units of packed RBCs, four units of fresh frozen plasma, 10 units of cryoprecipitate, and tranexamic acid. After rapid transfusion, blood pressure stabilized, and tachycardia improved. 

She was transferred to the ICU, where repeat hemoglobin improved to 16.3 g/dL. She developed agonal breathing, requiring intubation. Shortly after intubation, pulses were not palpable, and telemetry showed supraventricular tachycardia at approximately 150 beats per minute. Advanced cardiovascular life support (ACLS) was initiated for pulseless electrical activity (PEA) arrest. Cardiopulmonary resuscitation was performed for two cycles, and the patient received epinephrine and bicarbonate, after which a return to spontaneous circulation was achieved. Arterial blood gas obtained during the code was notable for a hemoglobin of 10.2 g/dL. General surgery recommended nonoperative management of the intra-abdominal hematoma after hemorrhage control was achieved. Follow-up CT demonstrated marked improvement in the hemoperitoneum, particularly in the pelvis, with residual simple ascites. The large mesenteric hematoma remained stable with embolization coils in place, and no new hemorrhagic process was identified.

The patient was extubated several days later and gradually improved clinically. Her abdominal pain decreased, and she was discharged home with home health services. At outpatient follow-up, she remained deconditioned with abdominal distension-related dyspnea, which had continued to improve. She was referred to general surgery for further discussion of the persistent hematoma and MALS-related celiac artery stenosis, with plans for continued outpatient monitoring.

## Discussion

This case demonstrates a rare and severe vascular complication of MALS-related celiac artery stenosis. The patient presented with acute abdominal pain and a large right-sided intra-abdominal hematoma but had no traditional risk factors for peripancreatic pseudoaneurysm formation, including pancreatitis, trauma, prior abdominal surgery, or recent intervention. The key vascular findings were a ruptured peripancreatic pseudoaneurysm arising from a celiac-SMA collateral vessel, severe proximal celiac artery stenosis, extensive collateralization, and a hook-shaped narrowing of the proximal celiac artery on sagittal imaging. Similar hemorrhagic presentations related to MALS and pancreaticoduodenal collateral vessels have been described in the literature [2,4,5,6]. 

Median arcuate ligament syndrome can cause focal narrowing of the proximal celiac artery, and CTA may demonstrate the characteristic focal narrowing or hook-shaped configuration of the celiac artery [1]. When celiac flow is reduced, collateral supply from the SMA through the pancreaticoduodenal arcade may increase [2,3]. This collateral pathway can become enlarged and tortuous, and chronic hemodynamic stress may contribute to pseudoaneurysm formation and rupture [2-4]. In this case, CTA and angiography demonstrated a peripancreatic pseudoaneurysm arising from a celiac-SMA collateral vessel in the setting of severe proximal celiac artery stenosis with extensive collateralization. The absence of common risk factors, including pancreatitis, trauma, infection, or prior abdominal surgery, supports altered collateral flow as the likely underlying mechanism [7,8].

The case also highlights the limitations of initial imaging. The first CT scan showed a large, complex collection with inflammatory stranding and only an indeterminate enhancing focus. This appearance could be mistaken for pancreatitis-related collection, infected fluid, bowel inflammation, appendicitis, or other intra-abdominal pathology. The diagnosis became clearer only after angiography, and repeat CTA demonstrated a pseudoaneurysm arising from a celiac-SMA collateral vessel. In a patient with unexplained intra-abdominal hematoma and falling hemoglobin, early repeat CTA should be considered even if the initial scan is not definitive, particularly because visceral pseudoaneurysms can deteriorate rapidly once bleeding occurs [7,9].

Management was challenging because of complex collateral inflow and outflow. The first angiographic attempt was unsuccessful because the feeding artery could not be identified or cannulated. Definitive management required a combined approach after standard transarterial cannulation failed. Direct percutaneous access allowed control of active extravasation with thrombin injection, followed by coil embolization of the jejunal collateral inflow and the superior and inferior pancreaticoduodenal artery pathways to exclude the pseudoaneurysm. Endovascular embolization is commonly used for visceral pseudoaneurysms and has been reported in similar MALS-related collateral vessel bleeding cases [4,5,6,8,9]. At the completion of the procedures, angiography confirmed the exclusion of the pseudoaneurysm while preserving major mesenteric and hepatic arterial flow.

The Society for Vascular Surgery (SVS) guidelines support treatment of visceral artery pseudoaneurysms because of their rupture risk, and endovascular therapy is commonly used when anatomically feasible [8,9]. In this case, direct percutaneous access was an important rescue technique after standard transarterial cannulation failed because of tortuous collateral vascular anatomy. Management of the underlying MALS-related celiac artery stenosis remains individualized. Long-term management decisions depend on symptoms after hemorrhage control, which is why immediate ligament release was not pursued in this patient. In reports of pancreaticoduodenal artery aneurysms (PDAAs) associated with MALS, some authors describe successful embolization alone, while others describe staged median arcuate ligament release or celiac revascularization to address persistent collateral flow [3-6]. In this patient, immediate hemorrhage control was the priority, and the hematoma was managed conservatively. Given persistent severe celiac stenosis and collateralization, outpatient follow-up with surgery/vascular surgery and interval CTA or duplex ultrasound would be reasonable to assess for persistent compression, recurrent pseudoaneurysm, or development of additional collateral vascular lesions [1,4-6].

Upon discharge, the patient followed up with general surgery for management of MALS and has not required any surgical intervention. She is yet to follow up with a vascular surgeon. She did have an episode of appendicitis approximately six months after her hospitalization out-of-state, which was treated conservatively with antibiotics.

## Conclusions

Ruptured peripancreatic pseudoaneurysm is a rare but life-threatening cause of intra-abdominal hemorrhage. In patients without pancreatitis, trauma, or prior surgery, severe celiac artery stenosis with celiac-SMA collateralization should be considered. A hook-shaped narrowing of the proximal celiac artery on sagittal imaging is characteristic of MALS. Initial CT may be nonspecific, and repeat CTA is important when anemia or clinical status worsens. Complex collateral pseudoaneurysms may require advanced endovascular techniques, including direct percutaneous access when transarterial cannulation fails, with thrombin injection as used for active extravasation and the use of coil embolization to exclude inflow and outflow vessels. Long-term imaging follow-up is important because persistent celiac stenosis may increase the risk of recurrent or new collateral vascular lesions.

## References

[1] Horton KM, Talamini MA, Fishman EK (2005). Median arcuate ligament syndrome: evaluation with CT angiography. Radiographics.

[2] Rebelos E, Cipriano A, Ferrini L, Trifirò S, Napoli N, Santini M, Napoli V (2019). Spontaneous bleeding of the inferior pancreatic-duodenal artery in median arcuate ligament syndrome: do not miss the diagnosis. Oxf Med Case Reports.

[3] Sgroi MD, Kabutey NK, Krishnam M, Fujitani RM (2015). Pancreaticoduodenal artery aneurysms secondary to median arcuate ligament syndrome may not need celiac artery revascularization or ligament release. Ann Vasc Surg.

[4] Chivot C, Rebibo L, Robert B, Regimbeau JM, Yzet T (2016). Ruptured pancreaticoduodenal artery aneurysms associated with celiac stenosis caused by the median arcuate ligament: a poorly known etiology of acute abdominal pain. Eur J Vasc Endovasc Surg.

[5] Casey L, Gananadha S, Jones A (2022). Ruptured pancreaticoduodenal artery aneurysm with median arcuate ligament compression: a two staged approach to management. EJVES Vasc Forum.

[6] Shimbara K, Shintakuya R, Honmyo N, Nakagawa N, Kohashi T (2023). Median arcuate ligament resection for a patient with ruptured pancreaticoduodenal artery aneurysm: a case report. Int J Surg Case Rep.

[7] Saad NE, Saad WE, Davies MG, Waldman DL, Fultz PJ, Rubens DJ (2005). Pseudoaneurysms and the role of minimally invasive techniques in their management. Radiographics.

[8] Madhusudhan KS, Venkatesh HA, Gamanagatti S, Garg P, Srivastava DN (2016). Interventional radiology in the management of visceral artery pseudoaneurysms: a review of techniques and embolic materials. Korean J Radiol.

[9] Chaer RA, Abularrage CJ, Coleman DM, Eslami MH, Kashyap VS, Rockman C, Murad MH (2020). The Society for Vascular Surgery clinical practice guidelines on the management of visceral aneurysms. J Vasc Surg.

